# Acceptability of Long-Acting Injectable Cabotegravir (CAB LA) in HIV-Uninfected Individuals: HPTN 077

**DOI:** 10.1007/s10461-020-02808-2

**Published:** 2020-02-12

**Authors:** Elizabeth E. Tolley, Sahar Z. Zangeneh, Gordon Chau, Joe Eron, Beatriz Grinsztejn, Hilton Humphries, Albert Liu, Marc Siegel, Maseko Bertha, Ravindre Panchia, Sue Li, Leslie Cottle, Alex Rinehart, David Margolis, Andrea Jennings, Marybeth McCauley, Raphael J. Landovitz

**Affiliations:** 1 Behavioral, Epidemiological & Behavioral Sciences, FHI 360, 359 Blackwell Street, Durham, NC 27701 USA; 2 Fred Hutchinson Cancer Research Center, Seattle, WA USA; 3 University of North Carolina at Chapel Hill, Chapel Hill, NC USA; 4 Evandro Chagas National Institute of Infectious Diseases, Oswaldo Cruz Foundation, Rio de Janeiro, Brazil; 5 Centre for the AIDS Programme of Research in South Africa, University of KwaZulu-Natal, Durban, South Africa; 6 Bridge HIV, Population Health Division, San Francisco Department of Public Health, San Francisco, CA USA; 7 School of Medicine and Health Sciences, George Washington University, Washington, DC USA; 8 UNC Project-Malawi, Lilongwe, Malawi; 9 Perinatal HIV Research Unit, Chris Hani Baragwanath Hospital, Soweto, South Africa; 10 ViiV Healthcare, Durham, NC USA; 11 Science Facilitation, FHI 360, Durham, NC USA; 12 Science Facilitation, FHI 360, Washington, DC USA; 13 UCLA Center for Clinical AIDS Research & Education, Los Angeles, CA USA

**Keywords:** HIV prevention, Clinical trial, Acceptability, PrEP, Injectable, Females, Males

## Abstract

Long-acting injectable PrEP could offer an alternative to daily oral PrEP, improve adherence and protection, if found acceptable, safe and effective. HPTN 077 evaluated injectable cabotegravir safety, tolerability and pharmacokinetics among HIV-uninfected males and females in sequentially-enrolled cohorts of two dosing strategies. We compared acceptability of product attributes, prevention preferences and future interest in injectable PrEP (FIIP) by region, sex-at-birth, arm and cohort and used multivariable analysis to identify FIIP determinants. Baseline injectable PrEP preferences were higher in non-U.S. sites and increased in both regions over time. In multivariable models, FIIP was most strongly associated with acceptability of product attributes, was higher in non-U.S. sites and more altruistic participants. Treatment arm and report of pain were not associated with FIIP. Injectable acceptability was highest in non-U.S. sites. Preferences for injectable versus other PrEP methods were higher among U.S. males than females, but higher among males and females in non-U.S. settings.

**Electronic supplementary material:**

The online version of this article (10.1007/s10461-020-02808-2) contains supplementary material, which is available to authorized users.

## Introduction

In the absence of an efficacious HIV vaccine, the need to develop and evaluate new biomedical HIV prevention products and methods of administration remains critical. In 2018, about 1.7 million people became newly infected with HIV; over two-thirds of new infections occurred in Africa and almost one-half occurred among key populations (including men who have sex with men (MSM), injection drug users, male and female sex workers, and transgender persons) and their partners [1, 2]. HIV infection rates vary substantially across different regions and population groups. In sub-Saharan Africa, most new infections (56%) occur in heterosexual women. Among individuals aged 15–24, more than two-thirds of new infections (67%) are in women [3]. In contrast, in 2016, 68% of all new infections in the U.S. were among MSM, with the highest rates of infection among black and Latino MSM [4].

Oral PrEP with tenofovir disoproxil fumarate and emtricitabine (TDF/FTC) efficacy has varied widely across a range of clinical trials and enrolled populations. Daily oral TDF/FTC PrEP reduced the risk of HIV infection by 75% among HIV serodiscordant heterosexual couples in Kenya and Uganda [5], but the same combined TDF/FTC regimen demonstrated a 44% reduction in HIV acquisition among MSM in a multi-country trial (iPrEx) [6], and no evidence of effectiveness in two large phase 3 trials in African women (FemPrEP and VOICE) [7, 8]. While differential tissue penetration of PrEP agents may account for some of the variability in PrEP efficacy [9], adherence appears to be one of the strongest predictors of efficacy [10]. Less than 40% of HIV-uninfected women on active product in FEM-PrEP and less than 30% in VOICE showed recent biomarker evidence of study product use [7, 8]. In an iPrEx sub-study, U.S. participants were significantly more likely than those from non-U.S. sites to report and demonstrate recent product use [11]. Inadequate adherence in PrEP trials has been associated with low HIV risk perception, non-acceptability of product-related attributes, alternative motivations for—and perceived stigma and non-disclosure of trial participation [12].

Although the Food and Drug Administration (FDA) approved TDF/FTC for oral PrEP in 2012, uptake in the U.S. has been low, especially among females and in black and Hispanic populations [13]. The delivery and uptake of oral TDF/FTC PrEP has been similarly slow in African settings [14]. And, while the cost of medication was initially a major barrier to PrEP uptake, other issues have emerged as greater challenges to expanded use of oral PrEP [15]. They include low levels of oral PrEP information and lack of integration of PrEP services into existing community-based and primary health systems that meet the needs of different population groups [16].

A long-acting injectable (LAI) product could address some of the challenges of taking a daily oral regimen [17]. Nevertheless, a LAI-PrEP could present other challenges to acceptability, including the need for frequent clinic visits (or potentially in the future, self-injection), or concerns related to the number and/or volume of injections, pain or the irreversibility of an injectable method [18]. To date, two different LAI-PrEP products (TMC278/rilpivirine [RPV LA] and GSK1265744/cabotegravir [CAB LA]) have been evaluated for safety and acceptability. Although RPV LA was found to be safe, generally well tolerated and acceptable to a cohort of African and U.S. women, the product is not currently being advanced for further evaluation as a prevention option, in part due to the need for cold chain storage and concerns about resistance [19, 20]. CAB LA is currently being evaluated in phase 3 trials after being first evaluated for safety, tolerability, and pharmacokinetics (PK) among U.S. men in the ECLAIR trial [21] and subsequently among HIV-uninfected, low-risk males and females in South Africa, Malawi, Brazil and the U.S. in HPTN 077 [22]. Several ECLAIR sub-studies shed light on participants’ experiences of pain and overall interest in injectable PrEP. Among 28 participants who received CAB LA injections, the reported experience of pain was highly variable, with about a third of participants reporting “no pain”, 38% reporting “minor” and 28% “minor or moderate” pain [23]. A qualitative study with the same cohort reported similar findings, noting that most participants preferred an injectable over other prevention options despite side effects, due to the “peace of mind” they experienced from its ease of use and duration of potential protection [24]. HPTN 077 expands on lessons from the ECLAIR phase 2a study and offers a unique opportunity to better understand how acceptability of a new prevention method might vary for different at-risk populations or by dosing strategy. Specifically, through our analyses of HPTN 077 acceptability data, we aimed to (1) assess acceptability of injectable product attributes; and (2) evaluate males’ and females’ future interest in using an injectable PrEP product. For both aims, we determine whether acceptability of specific attributes and future interest in using a LAI-PrEP product differ by sex, broad geography, or dosing strategies.

## Methods

HPTN 077 was a multi-site, randomized, double-blind, placebo-controlled Phase 2a trial to evaluate the safety, tolerability and acceptability of CAB LA. Primary study results on safety, tolerability, and PK have been previously described [22]. The study sequentially enrolled two cohorts evaluating distinct regimens of intramuscular gluteal injections; the first evaluated an 800 mg dose (delivered as two 2 mL injections) every 12 weeks over three injection cycles (C1); the second evaluated 600 mg (delivered as a single 3 mL injection) administered every 8 weeks, with the first two injections separated by 4 weeks (C2) over five total injections. In both cohorts, participants were randomized into active (CAB) or placebo arms in a ratio of 3:1. Prior to receiving injections, participants received 4 weeks of daily oral pills as a run-in period to assess any safety concerns to the active study product, and a 1-week washout. Participants randomized to placebo injections received a 4-week daily oral placebo tablet during the run-in period.

### Measures

#### Acceptability Measures

All participants were administered a baseline face-to-face acceptability questionnaire that assessed participants’ initial attitudes towards PrEP characteristics, including what they think they would like (e.g., nothing; HIV prevention; ease of use; long duration; discretion; offered by provider; no interruption of sex) and dislike (e.g., nothing; no HIV prevention; painful; side effects; no reversibility; offered by provider; no discretion; cost) about the method. Prompts to “likes” and “dislikes” were not read, but interviewers coded spontaneous responses into pre-established options or an “other” category. One week after the 1st, 2nd, 3rd (both cohorts) and 4th and 5th (C2 only) injection visit, participants responded to a questionnaire that assessed acceptability of five product attributes on a scale of 1 = highly unacceptable to 6 = highly acceptable. Product attributes included: receiving two (C1) or one (C2) injection at a visit; size/quantity of each injection; receiving injections every three (C1) or two (C2) months; injection site in the buttocks and degree of privacy. We also assessed the acceptability of three physical experiences of injection, also rated on a 6-point scale: pain at injection site; rash or reaction at injection site; and side effects experienced since last injection. Participants could indicate that they did not experience any pain, rash/reaction or side effects, in which case their acceptability rating related to the physical symptom was recoded from missing to 6 = highly acceptable. We used the average of the five items related to “product attributes” and the average of the three items related to “physical experiences” as two separate time-varying covariates.

Any injection site reactions (ISR) that were either reported or observed during study visits were recorded on a separate adverse event log. These ISRs were further graded by clinic staff as mild, moderate or severe. We included a count variable of the number of reported ISRs, regardless of grade, in this analysis.

#### Future Interest in Injectable PrEP

Two different outcomes were measured to assess future interest in injectable PrEP (FIIP). Trial participants’ preferences for HIV prevention, including condoms, oral, vaginal/rectal gel, ring or injectable PrEP options, were assessed 1 week after first and last injection visit (30 weeks for C1 and 34 weeks for C2). In addition, at 1 week after each injection visit, participants were asked how much they agreed with six statements (1 = disagree a lot to 6 = agree a lot) related to future use of PrEP injections, should results find the injection to be safe and at least partially effective in preventing HIV. We used one of the statements (“You would definitely use the injection for some time”) to assess FIIP.

#### Potential Determinants of FIIP

The baseline face-to-face acceptability questionnaire included questions about ever use of injections for contraception (for women) or other prevention or treatment purposes; HIV risk perception (not, somewhat or very worried), and past HIV prevention behaviors (nothing, monogamy, male/female condom use and/or HIV testing—multiple behaviors possible). In addition, participants were asked to rate their level of agreement with seven items describing their motivations for trial participation on a scale of 1 = disagree a lot to 6 = agree a lot. Motivations included altruistic reasons (e.g., “to help scientists”, “to help family and friends” and “to gain knowledge”) and other personal and health benefits (e.g., “doctor’s recommendation”, “personal risk”, “payment” and “access to healthcare”). The seven items pertaining to motivations for trial participation were summarized into two separate mean scores: the average of the three items related to “altruism” and the average of the four items related to “personal benefits”.

### Quantitative Analyses

We compared participants’ baseline socio-demographic and risk characteristics by sex-at-birth, site (U.S. and non-U.S.) and cohort, using Chi square or Fisher’s Exact tests for categorical variables, and t-tests for continuous variables.

#### Acceptability of Product Characteristics, Attributes and Physical Experiences

We compared characteristics of a PrEP injectable that participants liked and disliked by sex-at-birth, region and cohort, using Chi square tests. We depicted frequency tables of the five product attribute items and three physical experience items as stacked bar graphs in order to qualitatively assess and compare the reported levels of acceptability (from 1 = highly unacceptable to 6 = highly acceptable) for each item. We used t-tests to compare the mean score of product attribute and physical experience composite scores, as well as ISR count by sex-at-birth, region, cohort and arm.

Secondly, we assessed whether there was a significant association between perceived pain to time of discontinuation of the injectable study product using Cox proportional hazards model with time to permanent product discontinuation in the injection phase as the outcome, and the longitudinal measure of acceptability of pain as the independent variable.

#### Future Interest in Injectable PrEP

We performed both bivariate and multivariable analyses using a longitudinal ordinal logistic regression model to identify determinants of FIIP in our study population. We considered several baseline covariates, including an ordinal ‘HIV risk perception’ variable (not at all, somewhat or very worried) and two composite summary variables representing altruistic and personal motivations for trial participation. We examined the association of “ever use of injection for contraception” with FIIP among females-at-birth in the bivariate regression model, but not in the multivariable model including both sexes at birth. We also included continuous time-varying covariates describing ‘level of condom use in last month’ (never, rarely, sometimes, frequently or always used a condom), summary scores describing acceptability of ‘injectable attributes’ and “physical experiences” and the number of injection site reaction (ISR) adverse events (AEs) experienced prior to each acceptability questionnaire visit. Treatment arm, sex-at-birth, cohort and region (U.S. vs non-U.S.) were also included in the model. The models were fit via generalized estimating equations (GEE), assuming an independence covariance structure [25], using Proc Genmod procedure in SAS. All analyses were implemented using SAS software (version 9.4).

Finally, although the primary analysis made comparing between U.S. and non-U.S. regions, we also evaluated whether our bivariate and multivariable model findings differed when regional comparisons were re-defined as Americas vs Africa to reflect differences in most-at-risk populations—primarily MSM in the Americas (U.S. and Brazil) versus women in Africa (South Africa and Malawi.)

### Research Ethics

The HPTN 077 study protocol was approved by the designated ethics committee and/or institutional review board for each of the study sites; all participants provided written informed consent.

## Results

As shown in Table 1, a total of 199 participants enrolled across the two cohorts, with 110 participants in C1 (82 on CAB LA and 28 on placebo) and 89 participants in C2 (69 on CAB LA and 20 on placebo). Overall, 66% of participants were born female (n = 132) and 47% of all participants were from non-U.S. sites (n = 93). Regional differences at baseline included employment, prior injection experience, HIV risk perception and HIV testing as an HIV prevention behavior. Seventy-four percent of U.S. participants versus 37% of non-U.S. participants were employed either full- or part-time. Among females, only 14% of U.S. versus 76% of non-U.S. females had ever used an injectable contraceptive method. About twice as many non-U.S. participants as those from the U.S. were somewhat or very worried about their risk of HIV. Some differences in employment status and HIV risk perception also exist by sex-at-birth and cohort (Table 1).

**Table 1:** Baseline socio-demographic and other characteristics, by region, sex at birth and cohort

	Total (n = 199)				Total (n = 199)			Total (n = 199)		
	Regional comparisons				Sex at birth comparisons			Cohort comparisons		
	Overall (n = 199)	U.S. (n = 106)	Non-U.S. (n = 93)	P value	Male (n = 67)	Female (n = 132)	P-value	C1 (n = 110)	C2 (n = 89)	P-value
Age, median (IQR)	33.5	36.3	30.2	< 0.001	38	31.1	< 0.001	34.5	32.2	0.15
Sex at birth n (%)				< 0.001			–			0.77
Female	66	54	81		0	100		65	67	
Male	34	46	19		100	0		35	33	
Relationship status, n (%)				0.11			0.80			0.03
Married/civil union/legal partnership	25	24	26		24	25		20	30	
Living with primary partner	12	14	10		13	11		12	12	
Have primary partner, not living together	23	17	30		19	25		31	13	
Single/divorced/widowed	40	45	34		43	39		37	44	
Employment status (%)				< 0.001			< 0.001			0.03
Full-time employment	36	49	22		54	27		43	28	
Part-time employment	20	25	15		10	25		15	27	
Not employed	44	26	63		36	48		43	45	
Prior injection experience (%)										
Treatment	39	26	54	< 0.001	43	37	0.40	35	45	0.13
Prevention	68	87	47	< 0.001	70	67	0.70	62	76	0.03
Contraception (ever)	(n = 132) 49	(n = 57) 14	(n = 75) 76	< 0.001	(n = 0) 0	(n = 132) 49	–	(n = 72) 47	(n = 60) 52	0.60
HIV risk perception (%)				< 0.001			0.05			0.03
Not at all worried	55	70	39		49	58		59	51	
Somewhat worried	30	29	30		40	24		32	27	
Very worried	15	1	31		10	17		9	22	
Baseline prevention behaviors, current (%)										
Nothing	3	1	4	0.13	0	4	0.11	3	2	0.83
Monogamy	55	58	53	0.49	51	58	0.36	57	53	0.53
Male/female condom use	55	49	61	0.08	60	52	0.32	56	53	0.62
HIV testing	21	29	12	0.003	16	23	0.25	24	18	0.33
Motivations for trial participation (mean score, range 1–6)										
Altruism	5.1 (4.5, 6)	4.8 (4.3, 5.9)	5.4 (4.8, 6)	< 0.001	5.0 (4.4, 6)	5.1 (4.6, 6)	0.40	5.0 (4.3, 6)	5.2 (4.8, 6)	0.13
Personal & health benefits	3.5 (1.7, 5.2)	4.5 (2.9, 6.2)	2.4 (1, 3.4)	< 0.001	3.8 (2, 5.3)	3.4 (1.7, 5.2)	0.16	3.6 (1.7, 5.4)	3.4 (1.7, 5.2)	0.64

### Aim 1: Acceptability of Injectable PrEP Characteristics, Attributes and Physical Experiences

#### Baseline Attitudes Towards Injectable PrEP Characteristics

At baseline, participants most liked the idea that a PrEP injectable would be easier to use than other methods, might protect against HIV and could provide a longer duration of protection than other methods. However, about a third of participants expressed concerns about potential side effects and pain (Table 2).

**Table 2:** Baseline attitudes towards injectable PrEP characteristics liked and disliked, by region, sex at birth and cohort

	Total (n = 199)				Total (n = 199)			Total (n = 199)		
	Regional comparisons				Sex-at-birth comparisons			Cohort comparisons		
	Overall (n = 199)	U.S. (n = 106)	Non-U.S. (n = 93)	P-value	Male (n = 67)	Female (n = 132)	P-value	C1 (n = 110)	C2 (n = 89)	P-value
Liked characteristics (%)										
Nothing	2.5	2.8	2.1	0.76	3.0	2.3	0.76	3.6	1.1	0.26
HIV prevention	42.2	39.6	45.2	0.43	41.8	42.4	0.93	40.0	44.9	0.48
Easier to use	58.8	73.6	41.9	< 0.01	61.2	57.6	0.62	60.9	56.2	0.50
Long duration	47.7	54.7	39.8	0.04	37.3	53.0	0.04	50.9	43.8	0.32
Discreet	14.1	17.9	9.7	0.10	7.5	17.4	0.06	13.6	10.1	0.84
Offered by provider	12.6	19.8	4.3	< 0.01	9.0	14.4	0.27	12.7	4.5	0.94
No interruption of sex	13.6	20.7	5.4	< 0.01	11.9	14.4	0.63	15.4	5.6	0.39
Disliked characteristics (%)										
Nothing	24.1	14.1	35.5	< 0.01	20.9	25.8	0.45	24.5	23.6	0.88
No HIV prevention	12.1	14.1	9.7	0.33	13.4	11.4	0.67	16.4	6.7	0.04
Painful	36.2	40.6	31.2	0.17	34.3	37.1	0.70	33.6	39.3	0.41
Side effects	40.7	46.2	34.4	0.09	38.8	41.7	0.70	39.1	42.7	0.61
No reversal	12.1	17.0	6.4	0.02	9.0	13.6	0.34	15.4	7.9	0.10
Offered by provider	7.0	8.5	5.4	0.39	6.0	7.6	0.68	10.0	3.4	0.07
Not discreet	0.0	0.0	0.0	–	0.0	0.0	–	0.0	0.0	–
Cost	14.1	21.7	5.4	< 0.01	11.9	15.1	0.54	19.1	7.9	0.02

#### Acceptability of Product Attributes

**Fig. 1:**
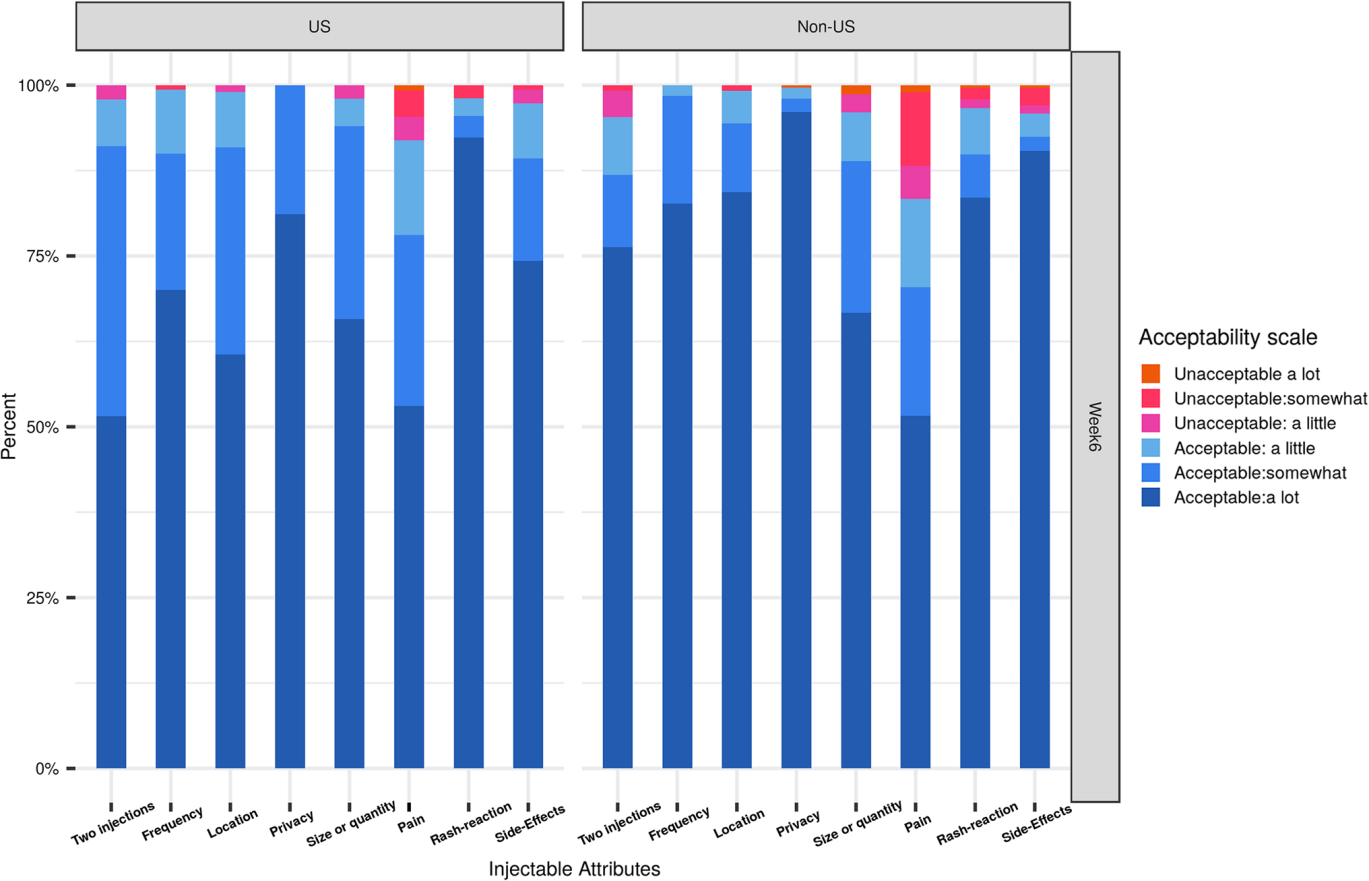
Acceptability of product attributes and physical experiences at week 6, by region and cohort 1

**Fig. 2:**
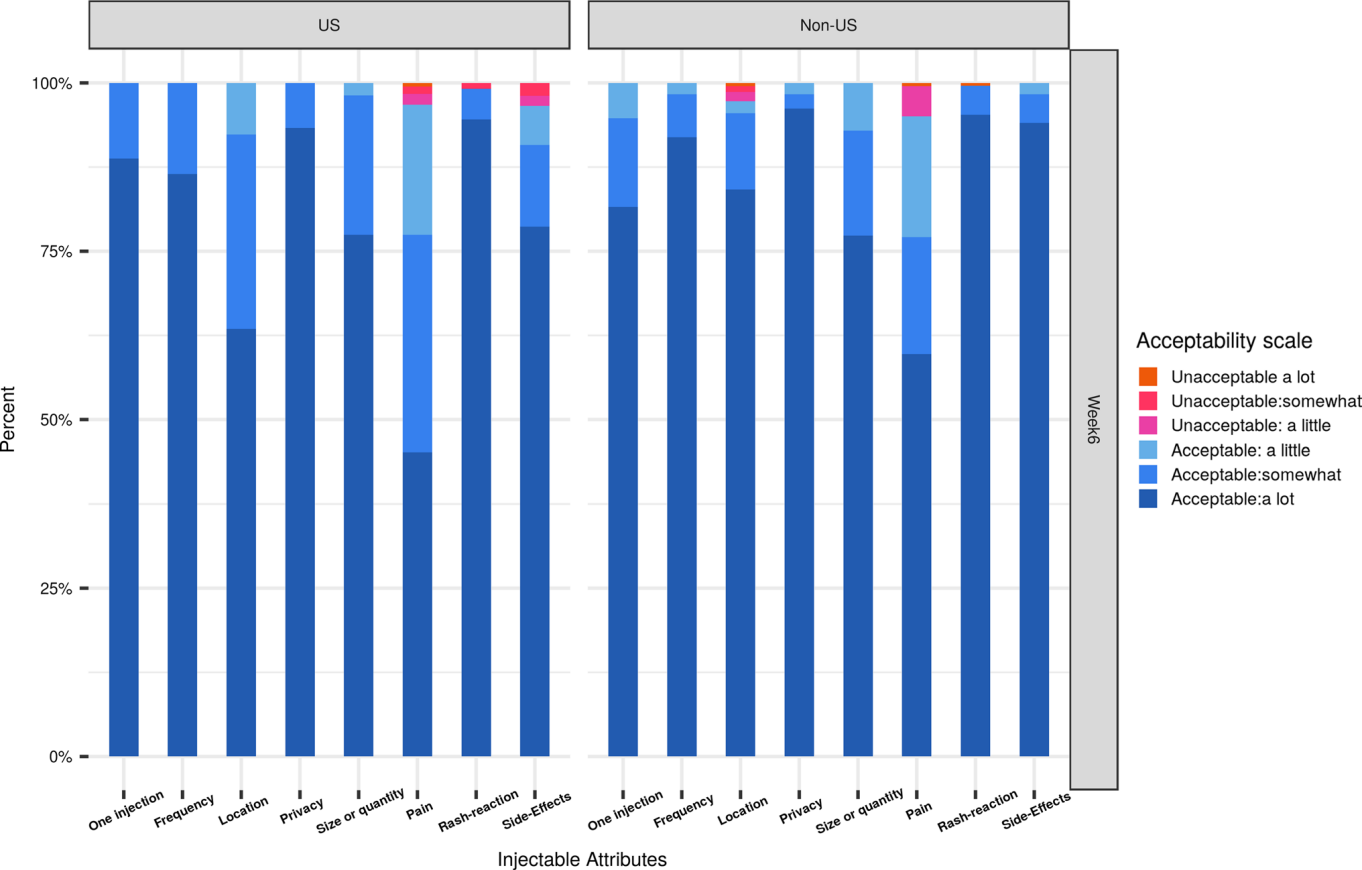
Acceptability of product attributes and physical experiences at week 6, by region and cohort 2

Participants’ acceptability of injectable attributes was high. After receiving a first injection at week 6, at least 50% of participants in C1 and approximately 75% or more of participants in C2 rated the number, frequency, location and duration of injection as highly acceptable (Figs. 1 and 2). Similar patterns of product attribute-related acceptability were reported at 1 week after last full injection (week 30 for C1 and 34 for C2) (Figs. S1 and S2 in Supplemental Materials). Overall, no significant difference was observed by region and visit. However, more non-U.S. participants reported pain to be unacceptable (a little, somewhat or a lot) than did U.S. participants at both timepoints. Participants receiving CAB LA injections were more likely to find the location of the injectable—in the buttocks—acceptable than those receiving saline injections (Figs. S3 and S4).

On the other hand, participants’ acceptability of physical experiences and their reported number of ISRs differed by cohort and arm. After their first injection, approximately 40% of C1 participants in the CAB LA arm and 75% of C1 placebo participants reported either experiencing no pain or pain that was highly acceptable (Fig. S3). This was similar for C2 participants (Fig. S4). After each injection visit, participants in the placebo arm reported significantly higher acceptability of physical experiences than those in the CAB LA arm. In addition, C2 versus C1 participants and those randomized to the placebo versus CAB LA arm reported significantly fewer ISRs across each timepoint (Table 3).

**Table 3:** Mean composite scores for product attributes, physical experiences and ISR counts over time, by region, gender, cohort and arm

	Total (n = 177)				Total (n = 177)			Total (n = 177)			Total (n = 177)		
	Regional comparisons				Sex at birth comparisons			Cohort comparisons			Arm comparisons		
	Overall (n = 177)	U.S. (n = 93)	Non-U.S. (n = 84)	P-value	Male (n = 60)	Female (n = 117)	P-value	C1 (n = 99)	C2 (n = 78)	P-value	CAB LA (n = 134)	Placebo (n = 43)	P-value
One week after 1st injection (week 6)													
Product attribute	5.6	5.6	5.6	0.64	5.6	5.6	0.39	5.5	5.7	0.002	5.6	5.6	0.74
Physical experiences	5.3	5.	5.3	0.79	5.5	5.2	0.07	5.2	5.5	0.01	5.1	5.8	< 0.0001
ISR count	1.2	1.1	1.3	0.42	1.0	1.3	0.11	1.4	1.0	0.04	1.6	0.2	< 0.0001
One week after 2nd injection (C1 week 18, C2 week 10)													
Product attribute	5.6	5.6	5.7	0.26	5.6	5.6	0.36	5.5	5.7	0.02	5.6	5.7	0.28
Physical experiences	5.4	5.4	5.4	0.96	5.4	5.4	0.99	5.4	5.5	0.32	5.3	5.7	< 0.0001
ISR count	1.2	1.2	1.2	0.97	1.2	1.2	0.74	1.4	0.9	0.02	1.6	0.2	< 0.0001
One week after 3rd injection (C1 week 30, C2 week 18)													
Product attribute	5.6	5.5	5.7	0.25	5.5	5.6	0.48	5.5	5.7	0.01	5.6	5.7	0.12
Physical experiences	5.4	5.4	5.4	0.65	5.5	5.3	0.12	5.3	5.5	0.21	5.3	5.8	< 0.0001
ISR count	1.4	1.4	1.4	0.98	1.2	1.5	0.16	1.7	1	0.002	1.7	0.3	< 0.0001
One week after 5th injection (C2 only week 34)													
Product attribute	5.5	5.5	5.6	0.58	5.5	5.6	0.58	–	5.5	–	5.5	5.6	0.78
Physical experiences	5.4	5.4	5.3	0.50	5.4	5.3	0.73	–	5.4	–	5.2	5.8	0.001
ISR count	0.8	0.9	0.8	0.93	0.6	1	0.17	–	0.8	–	1	0.3	0.0004

Scores range from 1 = unacceptable, a lot to 6 = acceptable, a lot

Product attribute acceptability scores remained high across study follow-up and were significantly higher in C2 than C1 participants, but did not differ by region, sex at birth, or arm. While composite scores for acceptability of physical experiences (i.e., injection site pain, rash and any side effects) were also generally high over time, participants’ scores were significantly higher after the first injection in C2 versus C1 and among those on placebo versus CAB LA over time.

Over the injection phase of the trial, 27 participants permanently discontinued injectable product use. Stated reasons for discontinuation included product-related side effects, inability or unwillingness to follow study procedures, abnormal lab values, reactive HIV tests, and desire for pregnancy or to terminate the study [20]. In a supplemental analysis, we found a significant inverse association between participants’ perceived acceptability of pain and discontinuation; participants with higher acceptability of pain had a lower risk for discontinuation. The risk of product discontinuation during the injection phase was associated with a 23% (hazard ratio 0.77, p = 0.02) reduction per one unit increase in participant’s acceptability of pain score.

### Aim 2: Future Interest in Using Injectable PrEP

#### Preferences for Prevention

At baseline, approximately half of U.S. participants, but almost three-fourths of non-U.S. participants (51% vs 71%) preferred an injectable HIV prevention method to other methods. In the U.S., preferences for a LAI-PrEP were higher among males than females. In non-U.S. sites, females were more likely to prefer an injectable PrEP compared to their male counterparts at baseline. At 1 week after last injection, (i.e., week 30 for C1 and week 34 for C2), 64% U.S. participants and 93% of non-U.S. participants preferred injectable PrEP to other methods (Table 4).

**Table 4:** Comparison of prevention preferences at baseline and last injection, by region and sex-at-birth

	Overall		Region			
	Baseline	Last Inj.	US		Non-US	
			Baseline	Last Inj.	Baseline	Last Inj.
Prevention preference (%)	(n = 198)	(n = 147)	(n = 105)	(n = 75)	(n = 93)	(n = 72)
No preference	4	1	6	1	1	0
2-monthly or 3-monthly injection	61	78	51	64	71	93
Daily oral pill	24	10	26	17	22	3
Vaginal ring	5	2	6	3	3	1
Vaginal (females)/rectal (males) gel	2	1	1	3	2	0
Other	6	7	10	12	1	1

	Male at birth				Female at birth			
	US		Non-US		US		Non-US	
	Baseline	Last Inj.	Baseline	Last Inj.	Baseline	Last Inj.	Baseline	Last Inj.
Prevention preference (%)	(n = 48)	(n = 40)	(n = 18)	(n = 14)	(n = 57)	(n = 35)	(n = 75)	(n = 58)
No preference	10	0	6	0	2	3	0	0
2-monthly or 3-monthly injection	56	68	67	93	47	60	72	93
Daily oral pill	29	25	28	7	23	9	20	2
Vaginal ring	n/a	n/a	n/a	n/a	11	6	4	2
Vaginal (females)/rectal (males) gel	2	3	0	0	0	3	3	0
Other	2	5	0	0	18	20	1	2

One missing observation

#### Determinants of Future Interest in Injectable PrEP (FIIP)

In univariate models (Table 5), FIIP was positively associated with non-U.S. versus U.S. region (OR 2.9, p = 0.0002), with higher levels of acceptability for product attributes (OR 4.77, p < 0.0001) and for physical experiences (OR 1.6, p = 0.0002), having higher levels of altruism (OR 1.96, p < 0.0001) and fewer total injection site reactions (OR 0.9, p = 0.004). In addition, C2 participants, who received one injection every 2 months, tended toward higher levels of FIIP than those in C1, who received two injections every three months (OR 1.64, p = 0.07). Females tended to have higher FIIP than males (OR 1.48, p = 0.16), as well as those with higher perceived HIV risk (OR 1.34, p = 0.13) and those with higher levels of baseline condom use (OR 1.12, p = 0.10), although these associations were not statistically significant. Among participants born female, having ever used a contraceptive injectable was associated with a significant increase in FIIP (OR 3.4, p = 0.001).

**Table 5:** Association of future interest in injectable PrEP (FIIP) with baseline characteristics, acceptability attributes and ISR

Parameter	Comparison	Bivariate results				Multivariable results			
		OR	95% CI		P-values	OR	95% CI		P-values
			LCL	UCL			LCL	UCL	
Cohort	2 vs 1	1.64	0.96	2.76	0.07	1.35	0.78	2.34	0.28
Sex at birth	Female vs. male	1.48	0.86	2.52	0.16	1.07	0.62	1.83	0.81
Region	Non-US vs. US	2.9	1.66	5.02	0.0002	2.9	1.63	5.16	0.0003
Treatment	Active vs. placebo	0.69	0.35	1.35	0.28	0.74	0.35	1.55	0.43
Condom use level		1.12	0.98	1.27	0.10	1.08	0.94	1.25	0.28
Worried-HIV		1.34	0.92	1.95	0.13	0.74	0.47	1.14	0.17
Product attributes		4.77	3.02	7.54	< 0.0001	4.84	3.09	7.59	< 0.0001
Physical experience		1.6	1.25	2.05	0.0002	1.04	0.82	1.3	0.77
Personal benefits		1.23	0.99	1.53	0.06	1.35	1.07	1.72	0.01
Altruism		1.96	1.47	2.61	< 0.0001	1.52	1.14	2.02	0.005
Total ISR count		0.9	0.83	0.97	0.004	0.98	0.9	1.07	0.65
Ever used injectable contraceptive	Yes vs no	3.4	1.61	7.20	0.001	n/a			

Among participants female at birth only

In the multivariable model, FIIP was most strongly associated with the composite acceptability score for product attributes (OR 4.84, p < 0.0001) (Table 5). Non-U.S. participants (OR 2.9, p = 0.0003) and those with higher levels of altruism at baseline (OR 1.52, p = 0.005) had higher FIIP. FIIP tended to be higher among participants in C2 than in C1. However, acceptability of physical experiences and number of reported ISRs were no longer significantly associated with FIIP in the multivariable model. In the sensitivity analysis, redefining the regional variable (Africa versus Americas) to more closely reflect differences in the epidemics, the multivariable model results were similar (Table SI). In this second model, African participants and those with higher product attribute acceptability reported higher FIIP as well as those with altruistic reasons for trial participation.

## Discussion

Adherence to daily oral tablet regimens remains one of the greatest challenges for successful PrEP use among a range of at-risk populations [8, 26, 27]. A LAI PrEP regimen could substantially improve PrEP coverage of sex acts, if those at risk for HIV acquisition were willing to initiate and continue product use. This analysis assessed the acceptability of two different injectable PrEP dosing regimens among a geographically diverse sample of participants in a phase 2a clinical trial (HPTN 077). It showed injectable CAB LA to be acceptable to both males and females in multiple geographic settings as a potential PrEP agent. It also provides some early indications of factors likely to affect demand of future injectable PrEP products should safety and efficacy be demonstrated in Phase 3 studies.

In general, participants found the injectable product to be somewhat to very acceptable. They preferred the idea of an injectable prevention method to other product delivery approaches prior to getting a first injection. After having received one or more injections, the proportion of participants preferring an injectable to other prevention methods was even greater. Participants who received a single injection every 2 months (C2) generally reported higher acceptability of product attributes and higher acceptability of physical experiences (e.g., pain or side effects), compared to those who received two injections every 3 months (C1). This is reassuring, as the pharmacokinetic data of the C2 regimen dictated that the dosing strategy using 600 mg every 8 weeks was the appropriate dose to move into pivotal Phase 3 trials. Overall, acceptability of product attributes—regardless of dosing regimen, was the strongest predictor of future interest in using an injectable PrEP product. Although lower acceptability of injection site pain was associated with discontinuation, these physical experiences were less important when other factors were accounted for in the multivariable model. Nevertheless, pain will likely be a factor for some individuals who initiate injectable PrEP use and provision of pain management strategies, including information, counseling and proactive use of topical or systemic pain medications could support continuation.

It is not surprising that future interest in using injectable PrEP was higher in non-U.S. than U.S. regions—and higher in African settings than the Americas, with some differences by sex-at-birth. These findings may be explained in part by geographic differences in the epidemic. Although the trial recruited participants at low risk for HIV by behavioral and biologic criteria [22], almost one-third of non-U.S. participants compared to just one percent of U.S. participants perceived themselves to be at a high risk for HIV acquisition. In the U.S. sites, males expressed higher interest than females in using a PrEP injectable, potentially reflecting the generally low perceived risk of U.S. females [28].

Both FIIP and ever use of injectable contraception were particularly high among females in non-U.S. settings, findings which were also reflected in phase 2 trial of RPV LA [20]. In several past HIV prevention trials of oral or topical products among African females, low adherence has been attributed to low HIV risk perception, females’ concerns about stigma, and challenges disclosing to partners compounded by the need to use a product daily [29–32]. Similar challenges have been reported to females’ use of contraception. Indeed, high rates of injectable contraceptive use in South Africa and other sub-Saharan countries are frequently attributed to both ease of use and discretion [33]. Clearly, females need prevention methods that are easier to use and may not require the cooperation or consent of male partners.

Introduction strategies, including how an injectable PrEP product is marketed and through which health systems it is delivered, will influence product demand and use. Mugo et al. suggest that delivery platforms should be aligned with the specific needs of each vulnerable population [14]. For females in African contexts where contraceptive injectables are widely used, provision through family planning or primary health clinics might prove easier than in U.S. settings. However, further implementation research will be needed to determine how best to co-deliver PrEP injectable and contraceptive options, including injectables, which may have different re-supply/re-injection schedules and procedures. Among MSM in U.S., an existing network of providers can be used [34], but may strain already burdened healthcare infrastructure. Nevertheless, current networks and screening approaches are likely to miss some higher risk and marginalized groups [35, 36]. In parallel with on-going phase 3 trials, additional research is needed to understand providers’ attitudes towards injectable PrEP, their perspectives on who might benefit from its use, and how to support optimal use [37, 38].

While this study suggests that acceptability of and demand for an injectable PrEP product will be high, several limitations should be considered. First, the data were collected in the context of a phase 2a clinical trial in which participants were at low risk for HIV infection by design and had to be willing to accept injections to be enrolled. Therefore, participants’ perspectives may or may not be similar to males and females who might seek to use injectable PrEP in routine health settings. Also, additional questions about the safety and effectiveness of CAB LA injectable PrEP remain that await Phase 3 trial evaluation. There are also uncertainties about the clinical significance of the prolonged “pharmacokinetic tail”.

Because the trial sequentially evaluated two different dosing regimens, it was possible to evaluate the effect of the size, number and frequency of injections on acceptability. However, the small sample size of this trial limited our ability to more fully examine differences in injectable PrEP acceptability by product, participant or regional characteristics. For example, although we found perceptions of pain influenced product continuation for some individuals, the small number of permanent discontinuations precluded us from evaluating the relative role of pain versus other reasons for stopping product use. Two fully powered phase 3 clinical trials, one in MSM and TGW (n = 4500) and one in African females (n = 3200) at high risk of HIV acquisition are currently ongoing and will provide critical information on safety, tolerability, and efficacy in at-risk populations. Furthermore, because these phase 3 participants randomized to receive either active CAB LA or daily oral TDF/FTC in a double-blind double-dummy study design, the trials may provide additional information on relative impact of ease of use, pain, or discretion on method use and whether/how acceptability of and adherence to PrEP modalities differ in higher risk populations.

## Conclusion

Long-acting injectable PrEP acceptability was high, especially at non-U.S. sites. Preferences for a LAI PrEP product compared to other methods was higher among males, many who were MSM, in the U.S., and higher among females in non-U.S. compared to U.S. sites, where both the need for new HIV prevention methods is greatest and contraceptive injectable use is widespread.

## Electronic supplementary material

Below is the link to the electronic supplementary material.

Supplementary material 1 (TIFF 25312 kb)

Supplementary material 2 (TIFF 25312 kb)

Supplementary material 3 (TIFF 25312 kb)

Supplementary material 4 (TIFF 25312 kb)

Supplementary material 5 (DOCX 23 kb)
