# Supplementary figures and images for: Acceptability of Long-Acting Injectable Cabotegravir (CAB LA) in HIV-Uninfected Individuals: HPTN 077
Source: AIDS Behav. 2020 Feb 12;24(9):2520–31. doi: 10.1007/s10461-020-02808-2 (PMC7423859; doi:10.1007/s10461-020-02808-2)

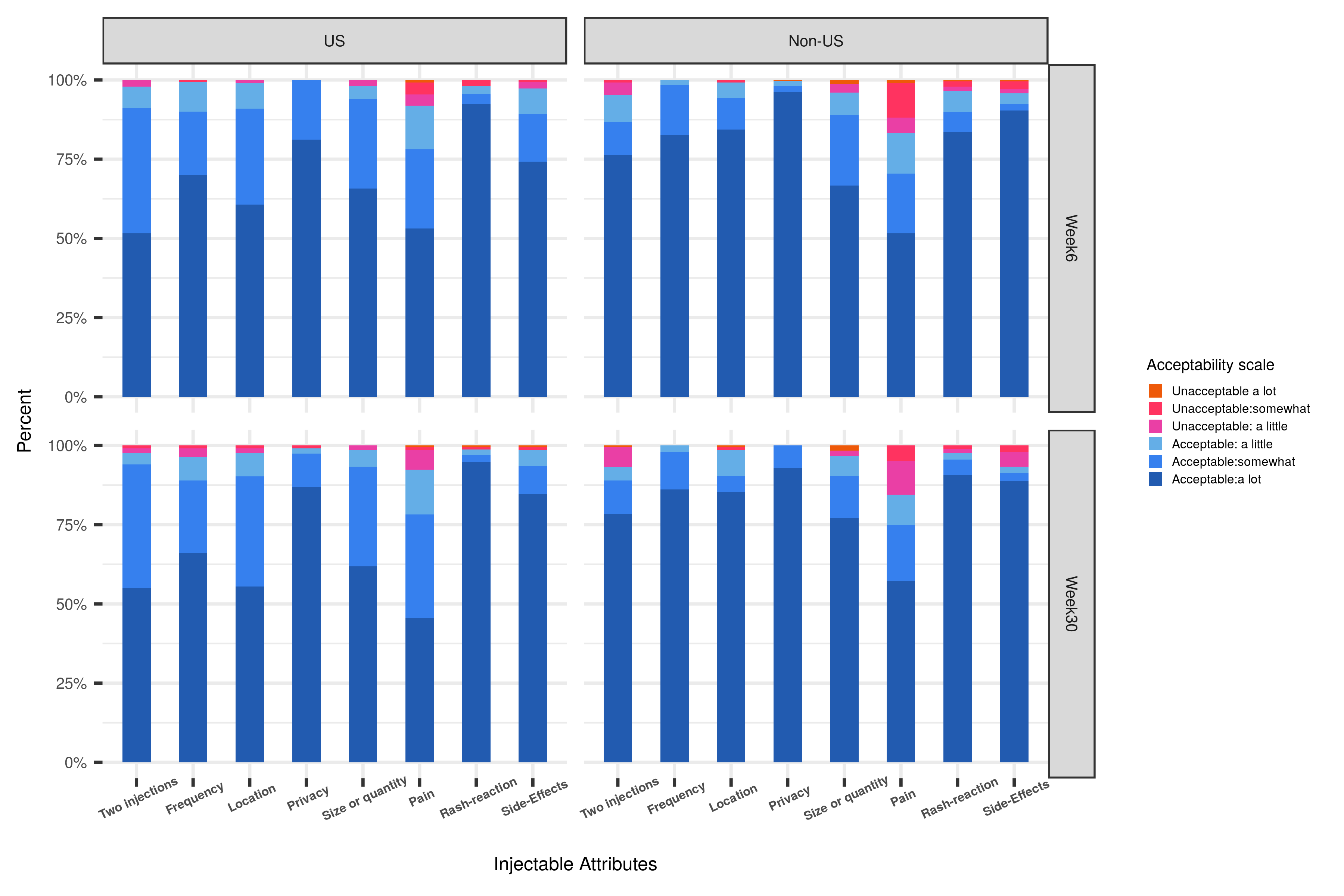

Supplement: Supplementary file 1 — Supplementary material 1 (TIFF 25312 kb) [file 10461_2020_2808_MOESM1_ESM.tif]

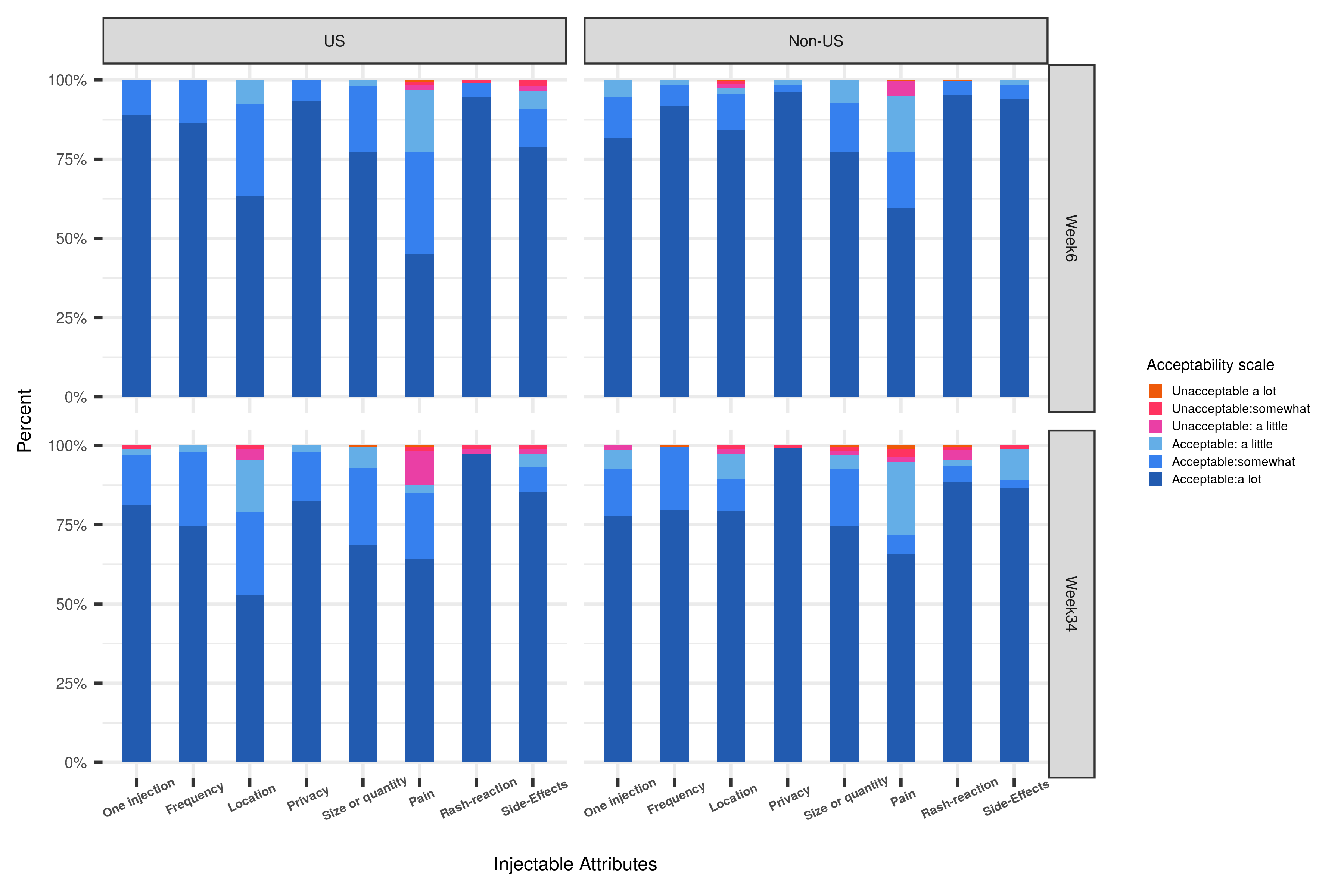

Supplement: Supplementary file 2 — Supplementary material 2 (TIFF 25312 kb) [file 10461_2020_2808_MOESM2_ESM.tif]

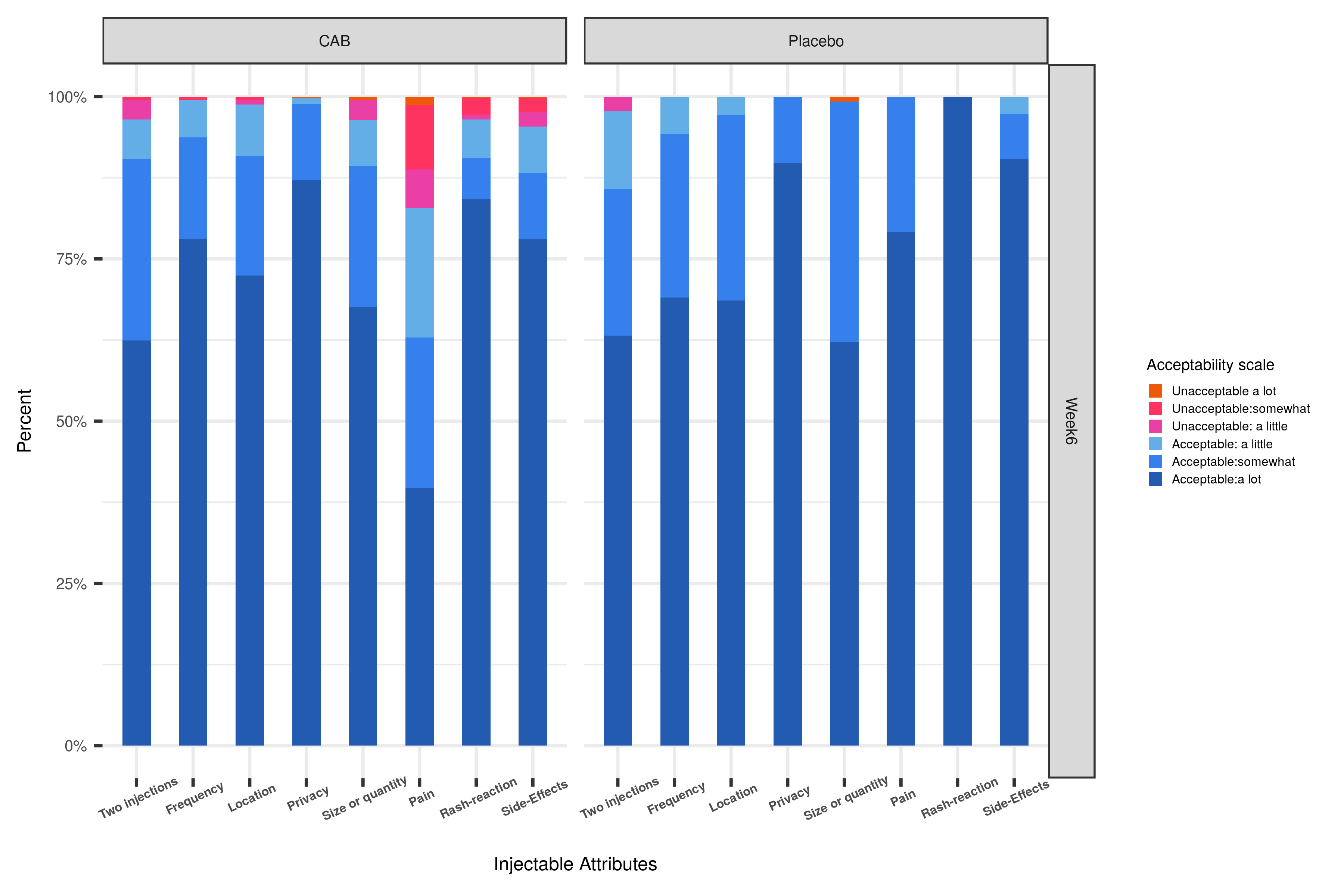

Supplement: Supplementary file 3 — Supplementary material 3 (TIFF 25312 kb) [file 10461_2020_2808_MOESM3_ESM.tif]

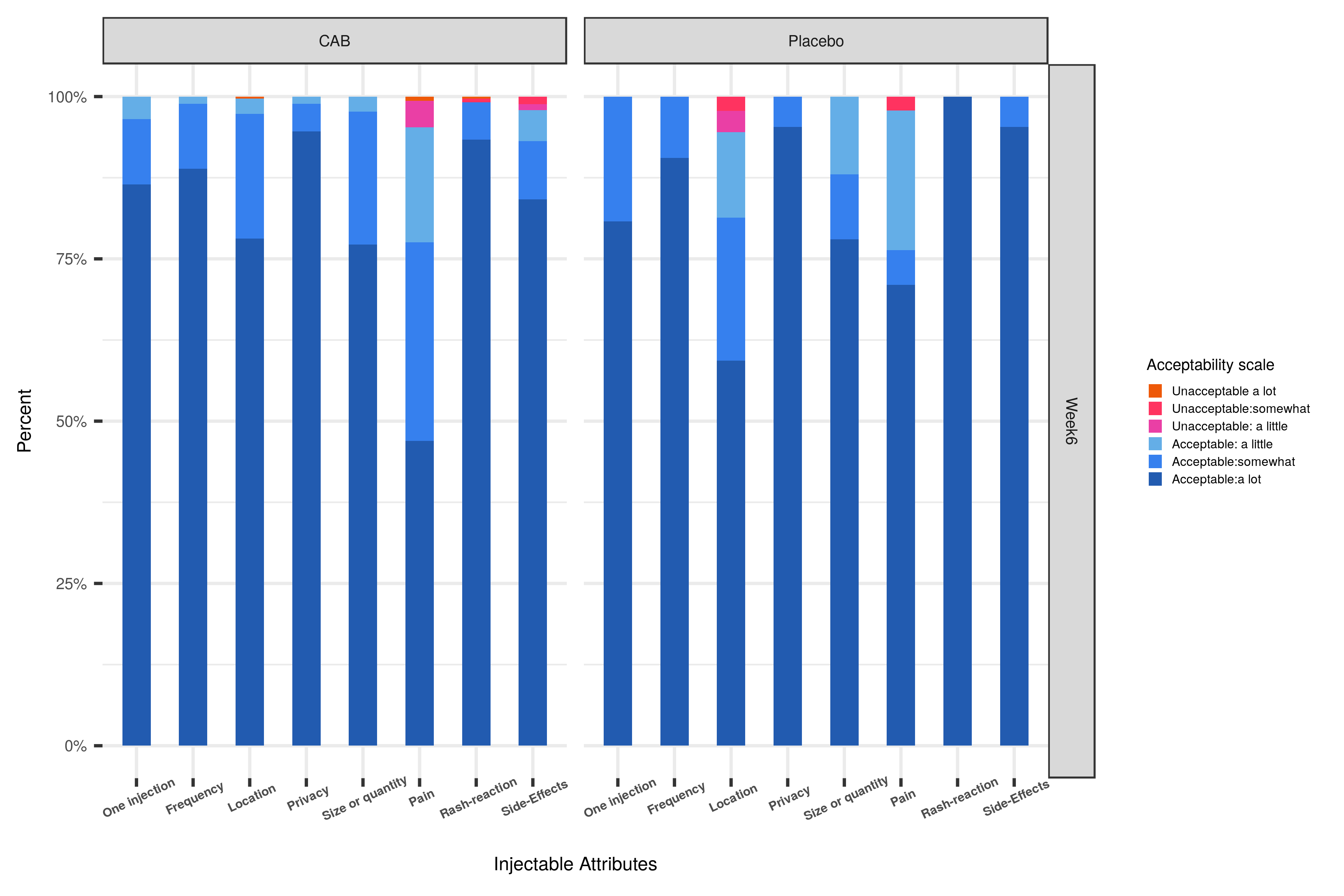

Supplement: Supplementary file 4 — Supplementary material 4 (TIFF 25312 kb) [file 10461_2020_2808_MOESM4_ESM.tif]
