# Supplementary material for: Acceptability of Long-Acting Injectable Cabotegravir (CAB LA) in HIV-Uninfected Individuals: HPTN 077
Source: AIDS Behav. 2020 Feb 12;24(9):2520–31. doi: 10.1007/s10461-020-02808-2 (PMC7423859; doi:10.1007/s10461-020-02808-2)
Supplement: Supplementary file 5 — Supplementary material 5 (DOCX 23 kb) [file 10461_2020_2808_MOESM5_ESM.docx]

Table SI. (Supplemental): Association of Future Interest in Injectable PrEP (FIIP) with Baseline Characteristics, Acceptability Attributes and ISR with redefined regions (Africa vs Americas)^d^

|  | | **Bivariate Results** | | | |  | **Multivariable Results** | | | |
| --- | --- | --- | --- | --- | --- | --- | --- | --- | --- | --- |
|  | | **95% CI** | | | |  | **95% CI** | | | |
| **Parameter** | **Comparison** | **OR** | **LCL** | **UCL** | **P-values** |  | **OR** | **LCL** | **UCL** | **P-values** |
| Cohort | 2 vs 1 | 1.63 | 0.96 | 2.76 | 0.07 |  | 1.33 | 0.77 | 2.32 | 0.31 |
| Sex at birth | Female vs. Male | 1.47 | 0.86 | 2.52 | 0.16 |  | 1.15 | 0.66 | 2.00 | 0.62 |
| Region 2 | Africa vs. Americas | 3.7 | 1.95 | 7.10 | 0.0001 |  | 2.73 | 1.40 | 5.32 | 0.003 |
| Treatment | Active vs. Placebo | 0.69 | 0.35 | 1.35 | 0.28 |  | 0.72 | 0.35 | 1.47 | 0.36 |
| Condom use level |  | 1.12 | 0.98 | 1.27 | 0.10 |  | 1.09 | 0.94 | 1.25 | 0.26 |
| Worried-HIV |  | 1.34 | 0.92 | 1.95 | 0.13 |  | 1.00 | 0.65 | 1.53 | 0.99 |
| Product attributes |  | 4.77 | 3.02 | 7.54 | <0.0001 |  | 4.70 | 2.99 | 7.39 | <0.0001 |
| Physical experience |  | 1.6 | 1.25 | 2.05 | 0.0002 |  | 1.05 | 0.82 | 1.34 | 0.71 |
| Personal benefits |  | 1.23 | 0.99 | 1.53 | 0.06 |  | 1.16 | 0.92 | 1.47 | 0.22 |
| Altruism |  | 1.96 | 1.47 | 2.61 | <0.0001 |  | 1.52 | 1.13 | 2.06 | 0.006 |
| Total ISR count |  | 0.9 | 0.83 | 0.97 | 0.004 |  | 0.99 | 0.92 | 1.08 | 0.85 |
| Ever used injectable contraceptive* | Yes vs No | 3.4 | 1.61 | 7.20 | 0.001 |  | n/a |  |  |  |
| ^d^Among participants female at birth only | | | | | | | | | | |
